# Laryngopharyngeal Reflux: The Last Decade

**DOI:** 10.3390/jcm11133592

**Published:** 2022-06-22

**Authors:** Petros D. Karkos, Jerome R. Lechien

**Affiliations:** 1 1st Academic ORL Department, AHEPA University Hospital, Aristotle University of Thessaloniki, 54124 Thessaloniki, Greece; 2 Laryngopharyngeal Reflux Study Group of Young-Otolaryngologists of the International Federations of Oto-Rhino-Laryngological Societies (YO-IFOS), Department of Otolaryngology-Head and Neck Surgery, Foch Hospital, School of Medicine, University Paris Saclay, 91190 Paris, France; jerome.lechien@umons.ac.be

Laryngopharyngeal reflux (LPR) and its consequences for the upper aerodigestive tract have been an issue of debate and controversy for more than three decades. From diagnosis to treatment, researchers and clinicians have been arguing on topics such as nomenclature, ideal diagnostics, pepsin, acid and alkaline components, symptom and finding questionnaires, ideal anti-reflux strategies and therapeutic duration. How do we define and, more importantly, how we treat something we “do not see” or “how do we treat symptoms with a multifactorial cause including reflux”?

There have also been many papers providing “exercises” in evidence-based medicine over the years, with all reaching completely opposite conclusions. A typical focus of several systematic reviews and metanalyses is the eternal question on the empiric treatment of LPR with proton pump inhibitors: does it work or not [1,2,3]?

In the last decade, there has been much emphasis on diagnostic algorithms for LPR, and this added to the disbelief in the classic strategy of empirical LPR treatment and whether this is still a valid treatment option 4. In the pediatric LPR world, it is even more crucial to reach a consensus on diagnosis and treatment, as the evidence of the link between severe upper respiratory problems, e.g., subglottic stenosis and acid, is strong.

The aim of this Special Issue (SI) is to highlight weaknesses in the way we approached atypical reflux patients in the past and, more importantly, to offer new theories, knowledge and, hopefully, evidence on how to change our perspectives on LPR diagnosis and treatment. Reflux questionnaires and a search for better and easier-to-use approach especially in primary care, impedance and ph-monitoring, as well as custom-tailored strategies beyond the classic PPI treatment for LPR, will be discussed in detail [4,5].

Hopefully, this SI will help clinicians to formulate a concise plan to approach the LPR patient in a more systematic way.
